# Empagliflozin Ameliorates Diabetic Cardiomyopathy via Attenuating Oxidative Stress and Improving Mitochondrial Function

**DOI:** 10.1155/2022/1122494

**Published:** 2022-05-09

**Authors:** Jinwu Wang, Xinyuan Huang, Hanjie Liu, Yuhang Chen, Peipei Li, Lingling Liu, Jiashen Li, Yangxi Ren, Junping Huang, Erya Xiong, Zhijie Tian, Xiaozhen Dai

**Affiliations:** ^1^School of Basic Medicine, Chengdu Medical College, Chengdu, China; ^2^Department of Pathology, West China Hospital, Sichuan University, Chengdu, China; ^3^School of Biosciences and Technology, Chengdu Medical College, Chengdu, China; ^4^School of Laboratory Medicine, Chengdu Medical College, Chengdu, China

## Abstract

Diabetic cardiomyopathy (DCM) is considered to be a critical contributor to the development of heart failure. Empagliflozin (EMPA), a sodium-glucose cotransporter 2 inhibitor, has been shown to prevent cardiovascular events and reduce the incidence of heart failure in randomized clinical trials. However, the mechanism of how EMPA prevents DCM is poorly understood. To study the potential mechanisms involved in the therapeutic effects of EMPA, we assessed the protective effects of EMPA on myocardial injury in type 2 diabetic *db/db* mice and H9C2 cardiomyocytes. 9–10-week-old male *db/db* mice were treated with EMPA (10 mg/kg) via oral gavage daily for 20 weeks. Afterward, cardiac function of treated mice was evaluated by echocardiography, and pathological changes in heart tissues were determined by histopathological examination and western blot assay. EMPA markedly reduced blood glucose levels, improved insulin tolerance, and enhanced insulin sensitivity of *db/db* mice. In addition, EMPA significantly prevented cardiac dysfunction, inhibited cardiac hypertrophy and fibrosis, and reduced glycogen deposition in heart tissues. Furthermore, EMPA improved diabetes-induced oxidative stress and mitochondrial dysfunction in both heart tissues of *db/db* mice and palmitate exposed H9C2 cells. EMPA significantly increased the expression of nuclear factor erythroid 2-related factor 2 (Nrf2) and its downstream genetic targets in cardiac tissue of type 2 diabetic *db/db* mice and H9C2 cells. EMPA also downregulated the expression of mitochondrial fission-related proteins and upregulated the expression of mitochondrial fusion-related proteins. Collectively, these findings indicate that EMPA may prevent DCM via attenuating oxidative stress and improving mitochondrial function in heart tissue.

## 1. Introduction

According to the International Diabetes Federation, diabetes is one of the fastest-growing chronic diseases worldwide and is projected to affect 693 million adults by 2045 [1, 2]. One of the major chronic complications associated with diabetes is diabetic cardiomyopathy (DCM) which affects approximately 70% of diabetic individuals. The clinical manifestations of DCM include left ventricular (LV) hypertrophy, myocardial fibrosis, and consequently impaired LV systolic and diastolic function, which eventually cause coronary artery disease, cardiac insufficiency, heart failure (HF), and sudden death [3].

Multiple mechanisms play direct and/or indirect causal roles in the development of DCM, including insulin resistance, glucotoxicity, lipotoxicity, impaired calcium handling, oxidative stress, mitochondrial dysfunction, and inflammation [3, 4]. Oxidative stress is considered to be one of the most pivotal factors involved in both the early and late stages of DCM [5]. In type 2 diabetes mellitus (T2DM), hyperglycemia, hyperlipidemia, hyperinsulinemia, and lipotoxicity result in the excess generation of reactive oxygen species (ROS) or reactive nitrogen species (RNS) and insufficient antioxidant capacity in the heart, thereby causing the pathogenesis of oxidative stress [6, 7]. Oxidative stress is detrimental to cardiac function and promotes the development of DCM by inducing cardiac cell death, cardiac hypertrophy, extracellular matrix (ECM) remodeling, and progressive cardiac fibrosis [8, 9]. Mitochondria are the powerhouses of cardiomyocytes and the main site for ROS production in cardiomyocytes [10]. Under physiological conditions, the excessive production of mitochondria-derived ROS can cause mitochondrial dysfunction, impair adenosine triphosphate (ATP) production in cardiomyocytes, and ultimately induce cell death and cause cardiac dysfunction [11]. In addition, oxidative stress regulates the morphology and function of mitochondria. Maintaining normal mitochondrial function is a prerequisite for providing energy to cells, but an imbalance between mitochondrial fusion and fission may cause excessive ROS production and result in dysfunctional mitochondria [12, 13]. Therefore, alleviating oxidative stress and improving mitochondrial function may be a potential strategy for preventing the development of DCM.

Common therapeutic options for T2DM focus on insulin, metformin, and sulfonylureas. However, there is no evidence these conventional hypoglycemic drugs reduce the incidence of cardiovascular disease in people with T2DM, and some hypoglycemic drugs may even increase the risk of HF [14–16]. Empagliflozin (EMPA) is a novel hypoglycemic agent that was approved for clinical use by the Food and Drug Administration on August 1, 2014 [17]. Unlike conventional hypoglycemic agents, EMPA lowers blood glucose levels by specifically inhibiting the activity of sodium-glucose cotransporter-2 (SGLT-2) in the S1 segment of the proximal renal tubule [18]. By doing so, treatment with EMPA results in excess blood glucose being transferred into the urine, thus lowering blood glucose levels in the body. The EMPA-REG OUTCOME trial demonstrated that treatment with EMPA reduces the risk of cardiovascular-related death by 38% in patients with T2DM with or without cardiovascular disease, and especially reduces the risk of all-cause mortality, cardiovascular mortality, and hospitalization (or readmission) for HF, and also decreases the probability of myocardial infarction or stroke [19]. The previous findings suggest that EMPA obviously improved cardiac function and ameliorated cardiac hypertrophy and fibrosis in different T2DM models [20–22]. Furthermore, a prospective observational study demonstrated that the protective effects of EMPA on LV dysfunction were more effective in the early stage of DCM than in advanced DCM, which means the early intervention of EMPA may be preferable for DCM [23]. These findings indicate promising potential for the application of EMPA in the treatment and/or reversal of DCM. However, the mechanisms of cardioprotective effects of EMPA are still not clear.

Here, we aimed to determine the potential protection of EMPA against myocardial injury in type 2 diabetic *db/db* mice and investigate the underlining mechanisms. In addition, we studied the effects of EMPA treatment on diabetic-like cardiac damage *in vitro* on the palmitate- (PA-) exposed H9C2 cells. These findings demonstrated that EMPA significantly improved cardiac function and inhibited cardiac hypertrophy and fibrosis via attenuating oxidative stress and improving mitochondrial function. Furthermore, EMPA attenuates oxidative stress and improves mitochondrial function mainly through activating the nuclear factor erythroid 2-related factor 2 (Nrf2) signaling pathway.

## 2. Materials and Methods

### 2.1. Animal Procedure and Drug Treatment

9–10 week-old diabetic *db/db* male mice (BKS.Cg-Dock7m+/+Leprdb) and littermate C57BLKS/J wild type (WT) mice were purchased from the Model Animal Research Center of Nanjing University (Nanjing, China). All mice were raised in a 12: 12-h light-dark cycle and specific pathogen-free conditions in the animal facility at Chengdu Medical College (Chengdu, China). All animal experiments were approved by the animal ethics committee of Chengdu Medical College. *db/db* mice were randomized to treatment either with EMPA via oral gavage (*db/db* + EMPA group), or treatment with oral gavage vehicle in the same dosing frequency (*db/db* group). Mice in the EMPA treatment group were administered EMPA (Boehringer-Ingelheimat, Germany) at a dose of 10 mg/kg/day for 20 weeks via oral gavage. WT and *db/db* group mice were both gavaged with 0.5% hydroxyethylcelluloseas vehicle with the same administration regimen. Since diabetic cardiomyopathy is a chronic disease, we chose to administer EMPA at a relatively low dose of 10 mg/kg/day over a relatively long duration of treatment (20 weeks) to study the long-term effects of EMPA on the development of cardiac complications associated with diabetes [21].

### 2.2. Cell Culture and Treatment

The heart myoblast cell line (H9C2 cells) was obtained from the Shanghai Institute of Biochemistry and Cell Biology (Shanghai, China) and was cultured in high glucose Dulbecco's modified Eagle's medium (DMEM; Gibco, Rockville, MD, USA), containing 10% fetal bovine serum (Gibco). Increased levels of plasma nonesterified fatty acids (NEFAs) occur in both obesity and T2DM, and PA is the most abundant plasma saturated fatty acid [24]. To study the effects of EMPA on the elevated levels of NEFAs that occur in obesity and T2DM, H9C2 cells were exposed to PA to mimic the cellular metabolic environment of obesity and type 2 diabetes. PA stock solution (10 mM) was prepared by dissolving sodium palmitate (Sigma-Aldrich, St. Louis, MO, USA) in 50% ethanol solution mixed with 2% fatty acid-free bovine serum albumin (BSA) solution, as previously described [25]. H9C2 cells were exposed to 200 *μ*M PA under a range of concentrations of EMPA (Selleck, Houston, TX).

### 2.3. Oral Glucose Tolerance Test and Intraperitoneal Injection Insulin Tolerance Test

Mice were fasted overnight and then treated with either oral glucose (1 g glucose/kg body weight) for oral glucose tolerance test (OGTT) procedures, or intraperitoneal insulin (1 U insulin/kg body weight) for intraperitoneal injection insulin tolerance test (IPITT) procedures. Blood glucose level of each mouse was measured at the stated time points using a glucometer and blood sampled from the tail vein.

### 2.4. Echocardiographic Evaluation

After the 20-week EMPA treatment, we used an ultrasound diagnostic apparatus (GE Vivid 7 Dimension) equipped with an i13L probe (12.0 MHz, 1.5 cm) to perform echocardiography on the experimental mice. Two dimensionally guided M-mode images of the parasternal short axis papillary muscle were collected. The following structural variables were assessed: heart rate (HR), interventricular septal width during end-diastole (IVSd), LV internal diameter at end-diastole (LVIDd), LV posterior wall diameter (LVPWd), LV internal diameter at end-systolic (LVIDs), LV end-diastolic velocity, LV end-systolic velocity, LV fractional shortening (LVFS), LV ejection fraction (LVEF), stroke volume, and cardiac output (CO).

### 2.5. Detection of Plasma Lipids

Fasting blood was collected into anticoagulant tubes containing EDTA and centrifuged at 3000 rpm for 15 min. Mouse plasma was collected. Total triglyceride (TG), total cholesterol (TC), high-density lipoprotein cholesterol (HDL-C) content, and low-density lipoprotein cholesterol (LDL-C) were detected by Elisa kits (Jiancheng Bioengineering, Nanjing, China), respectively, according to the manufacturer's manuals.

### 2.6. Histological Staining and Immunohistochemical Analysis

Mouse heart tissues were fixed overnight in 4% paraformaldehyde, then dehydrated, embedded in paraffin, and sectioned at 5 *μ*m thickness. To evaluate pathological changes, paraffin sections of heart tissues were stained with hematoxylin and eosin (H&E; Keygen Biotech, China). Sirius-red staining was used to detect heart tissue fibrosis. In addition, periodic acid-Schiff (PAS) staining was used to evaluate glycogen accumulation in heart tissues. Furthermore, we assessed myocyte cross-sectional area (CSA) by staining sections with fluorescein-conjugated wheat germ agglutinin (WGA; Alexa Fluor-488, Invitrogen, Carlsbad, CA). Images of sections were collected using a confocal microscope (Eclipse-TI2, Nikon, Japan).

For immunohistochemical analysis, heart tissue sections were dewaxed, hydrated, and then were subjected to antigen retrieval using 1× target retrieval solution (Dako, Carpinteria, CA). After antigen retrieval, sections were incubated with 3% hydrogen peroxide for 30 min and then blocked in 5% BSA for 30 min. The following primary antibodies were then applied overnight at 4°C: transforming growth factor-*β* 1(TGF-*β*1; 1: 400 dilution; #ab92486, Abcam, Cambridge, MA), Superoxide Dismutase 2 (SOD2; 1 : 400 dilution; #D3X8F, Cell Signaling Technology, Danvers, MA), Nuclear factor erythroid 2-related factor 2 (Nrf2; 1 : 200 dilution; #ab31163, Abcam, Cambridge, MA), Optic Atrophy 1 (OPA1; 1 : 400 dilution; #ab42364, Abcam, Cambridge, MA), mitofusin 1 (MFN1; 1 : 400 dilution; #ab57602, Abcam), and dynamin–related protein 1 (DRP1; 1 : 400 dilution; #NB110-55288, Abcam, Cambridge, MA). Subsequently, sections were incubated with the corresponding horseradish peroxidase-conjugated secondary antibodies (1 : 500 dilutions, Bioss Biotechnology, Beijing, China) for 1 h at room temperature. After rinsing three times in PBS, sections were incubated with Diamino-benizdine (DAB, Solarbio, China) and counterstained with hematoxylin (Solarbio, China).

### 2.7. Dihydroethidium Fluorescence Staining

To evaluate effects of EMPA on oxidative stress in heart tissues and PA-treated H9C2 cells, dihydroethidium (DHE) (Molecular Probes, Eugene, OR, USA) superoxide anion fluorescent probe staining was performed to evaluate the oxidative stress level of frozen heart sections and H9C2 cells, as described in our previous research [26]. Briefly, frozen heart sections of the left ventricle were incubated with 5 *μ*M DHE in a dark and humidified container at 37°C for 30 min. Then, the cell nuclei were stained by using 4′,6-diamidino-2-phenylindole (DAPI; Solarbio, China). After staining, fluorescence images were observed and collected using the BX63 Olympus fluorescence microscope (Olympus, Tokyo, Japan). H9C2 cells were seeded into 24-well plates and incubated for 24 h before treatment. Then, cells were exposed to 200 *μ*M PA in the presence of different concentrations (100 nM, 250 nM, and 500 nM) of EMPA for 24 h. H9C2 cells were subsequently incubated with DHE (5 *μ*M) at 37°C for 30 min. Following incubation, H9C2 cells were harvested and differences in fluorescence intensity were evaluated by flow cytometry (Aglilent Technologies Inc., Palo Alto, CA). The data was analyzed by FlowJo software (TreeStar, Ashland, OR).

### 2.8. Measurement of Mitochondrial Superoxide Formation

The Mito-SOX red mitochondrial superoxide indicator (Thermo Fisher, Waltham, MA) was used to detect the mitochondrial ROS (Mito-ROS) in heart tissues and H9C2 cells. In brief, frozen heart tissue sections were incubated with 5 *μ*M Mito-SOX at 37°C for 30 min, subsequently incubated with DAPI to stain the cell nuclei. As the staining in H9C2 cells, H9C2 cells were exposed to 200 *μ*M PA and treated with different concentrations (100 nM, 250 nM, and 500 nM) of EMPA for 24 h. Then, H9C2 cells were rinsed twice with PBS and incubated in 5 *μ*M Mito-SOX for 30 min at 37°C. Afterward, the cell nuclei were stained by DAPI. After staining, images were taken under the fluorescence microscope (BX63; Olympus, Tokyo, Japan).

### 2.9. Immunofluorescence Assay

The expression and location of Nrf2 in H9C2 cells after exposure to 200 *μ*M PA under different concentrations (100 nM, 250 nM, and 500 nM) of EMPA for 24 h were detected by immunofluorescence staining with anti-Nrf2 antibody (1 : 200 dilution; #ab31163, Abcam, Cambridge, MA). H9C2 cells were incubated with anti-Nrf2 antibody at 4°C overnight and followed by incubating with an Alexa fluorescein-labeled secondary antibody (Santa Cruz, Dallas, TX) for 1 h at 37°C. Nuclei were counterstained with DAPI. The stained images were taken using a fluorescence microscope (Olympus, Tokyo, Japan).

### 2.10. Western Blot Analysis

Western blot assays were performed to examine the expression of various proteins of interest. Heart tissues and cells were lysed in RIPA buffer (Beyotime Inc., Shanghai, China). Protein extracts were separated by sodium dodecyl sulfate polyacrylamide (SDS-PAGE) gel electrophoresis and transferred onto polyvinylidene fluoride (PVDF) membranes (Millipore, Billerica, MA). After blocking with 5% nonfat milk and 0.5% BSA in Tris-buffered saline with Tween®20 (TBST) for 1 h, membranes were incubated with primary antibodies overnight at 4°C. Then, membranes were washed with TBST for 3 times and incubated with corresponding secondary antibodies for 1 h at room temperature. The primary antibodies used to target proteins were as follows: SOD2 (1 : 1000 dilution; #D3X8F, Cell Signaling Technology, Danvers, MA), Nrf2 (1 : 1000 dilution; #ab31163, Abcam, Cambridge, MA), NADPH Quinone Oxidoreductase 1 (NQO1; 1 : 1000 dilution; #ab34173, Abcam, Cambridge, MA), 3-Nitrotyrosine (3-NT; 1 : 1000 dilution; #AB5411, Millipore), 4-hydroxynonenal (4-HNE; 1 : 1000 dilution; #HNE11-S, Alpha Diagnostic International, San Antonio, TX, USA), Optic Atrophy 1 (OPA1; 1 : 1000 dilution; #ab42364, Abcam, Cambridge, MA), MFN1 (1 : 1000 dilution; #ab57602, Abcam, Cambridge, MA), DRP1 (1 : 1000 dilution; #NB110-55288, Abcam, Cambridge, MA), and *β*-actin (1 : 3000 dilution, Bioss Biotechnology, Beijing, China). All secondary antibodies conjugated with horseradish peroxidase (HRP) were obtained from Bioss Biotechnology (Beijing, China). Blots were visualized with an enhanced chemiluminescence HRP substrate (Millipore). The amount of the protein was quantified using Image Lab analysis (Bio-Rad, Hercules, CA) and normalized against their respective controls.

### 2.11. Transmission Electron Microscope (TEM) Observation

Tissue specimens were fixed in 2.5% glutaraldehyde solution at 4°C and repaired with 1% osmium tetroxide. Tissue specimens were dehydrated and then embedded in epoxy resin. Slides mounted with 50 nm sections were incubated with lead citrate or uranium acetate and observed under a Hitachi H600 electron microscope (Hitachi, Japan).

### 2.12. Mitochondrial Morphology Staining

H9C2 cells were seeded in confocal dishes at the density of 1 × 10^5^ cells/dish and treated with 200 *μ*M PA in the presence of different concentrations (100 nM, 250 nM, and 500 nM) of EMPA for 24 h. After rinsing with PBS for three times, H9C2 cells were incubated with 200 nM MitoTracker® Red CMXRos (Thermo Fisher) for 30 min at 37°C. Mitochondrial images were taken using a confocal laser scanning microscope (Eclipse-TI2, Nikon, Japan).

### 2.13. Mitochondrial Membrane Potential

Mitochondrial membrane potential was assessed using a tetramethylrhodamine ethyl ester (TMRE) probe (Thermo Fisher). H9C2 cells were seeded in 96-well plate one day before treatment, and then, cells were exposed to 200 *μ*M PA in the presence of different doses (100 nM, 250 nM, and 500 nM) of EMPA for 24 h. Then, the cell culture medium was removed and replaced with serum-free medium containing 100 nM TMRE incubating for 20 min at 37°C. After washing three times with PBS, the fluorescence intensity was examined at 549 (excitation) and 576 nm (emission) wavelengths with a microplate reader (VICTOR® Nivo™, perkinelmer, MA).

### 2.14. Statistical Analysis

All data are presented as mean ± standard deviation (SD). Group differences were determined by the One-way analysis of variance using Graphpad Prism version 8.0 software. *P* values of <0.05 were considered statistically significant.

## 3. Results

### 3.1. Effects of EMPA on Metabolic Parameters of *db/db* Mice

**Figure 1:**
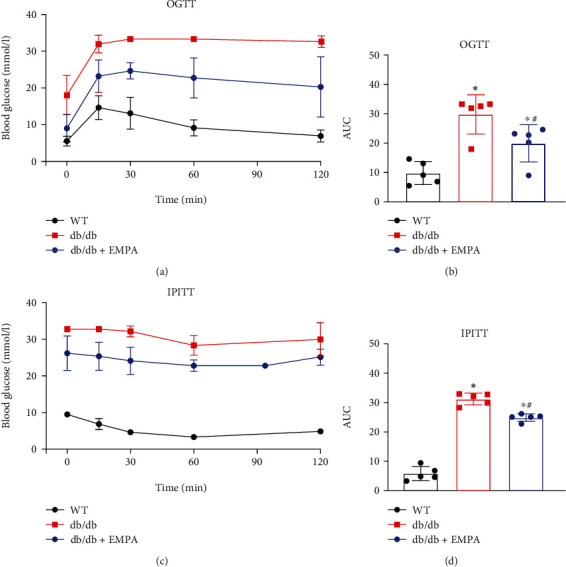
The effects of EMPA on glucose tolerance and insulin resistance of *db/db* mice. (a and b) The oral glucose tolerance (OGTT) and (c and d) intraperitoneal insulin tolerance test (IPITT) were performed in WT mice treated with vehicle and *db/db* mice treated with EMPA or vehicle for 20 weeks. Area under the curve (AUC) for each test is shown in b and d. *n* = 5 per group. Data are shown as the mean ± SD, ^∗^*P* < 0.05 vs. WT mice group; ^#^*P* < 0.05 vs*. db/db* mice treated vehicle group.

Diabetes-related biochemical assessments for the three experimental groups of mice are shown in Table 1. Compared with WT mice, *db/db* mice treated with vehicle had higher average body weights and heart weight/tibial length ratios. Compared to *db/db* mice in vehicle, *db/db* mice treated with EMPA had slightly lower average body weights and heart/tibial length ratios, but these values were not significantly different compared with *db/db* mice (*P* > 0.05). Both the fasting and nonfasting blood glucose levels of *db/db* mice treated with either vehicle or EMPA were obviously higher than those in the WT group. After treatment for 20 weeks, nonfasting blood glucose in *db/db* mice treated with EMPA was lower than in *db/db* mice treated with vehicle. In addition, the plasm TC, LDL-C, and TG were significantly higher in *db/db* groups than in WT mice (*P* < 0.05). However, *db/db* mice receiving EMPA treatment had significantly lower TC but not TG levels than *db/db* mice receiving vehicle. In addition, *db/db* mice receiving EMPA treatment had significantly higher HDL-C levels than *db/db* or WT mice receiving vehicle. The effects of EMPA on glucose tolerance and insulin resistance in *db/db* mice were evaluated by OGTT and IPITT assay, respectively. During OGTTs, blood glucose levels in *db/db* mice were markedly higher than those of WT mice at all time points, with greater areas under the curve (AUC). EMPA treatment significantly improved the glucose tolerance in *db/db* mice, as shown by the better glucose disposal measures and lower AUC in *db/db* mice treated with EMPA group (Figures 1(a) and 1(b)). During IPITTs, *db/db* mice showed greater insulin resistance, manifested by a reduced hypoglycemic effect of insulin and increased AUC compared to WT mice (*P* < 0.05). However, in EMPA-treated *db/db* mice, the AUC during IPITT was obviously lower than in *db/db* mice treated with vehicle, suggesting that EMPA improves insulin tolerance and enhances insulin sensitivity in *db/db* mice (Figures 1(c) and 1(d)).

### 3.2. EMPA Prevents Cardiac Dysfunction in *db/db* Mice

**Figure 2:**
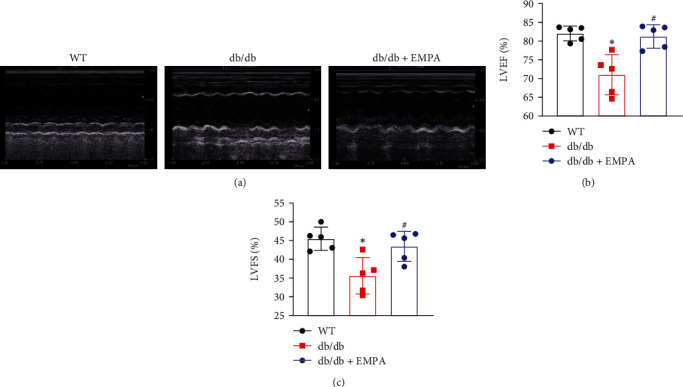
EMPA improved cardiac function of *db/db* mice. (a) Representative echocardiography images. (b) The values of left ventricular ejection fraction (LVEF) in each group of mice. (c) The values of left ventricular fractional shortening (LVFS) in each group of mice. *n* = 5 per group. Data are expressed as mean ± SD, ^∗^*P* < 0.05 vs. WT mice group; ^#^*P* < 0.05 vs. *db/db* mice treated vehicle group.

Echocardiographic assays were performed to determine whether EMPA treatment improved cardiac function of *db/db* mice (Figure 2 and Table 2). The results demonstrated that cardiac function in *db/db* mice was impaired, reflected by a lower HR, LVEF, and LVFS and higher LVIDd and LVIDs than WT mice. In contrast, *db/db* mice treated with EMPA had a significantly better cardiac function, with higher LVEF and LVFS scores, than that in *db/db* mice in the vehicle group.

### 3.3. EMPA Inhibited Diabetes-Induced Pathological Changes in Myocardial Tissue

**Figure 3:**
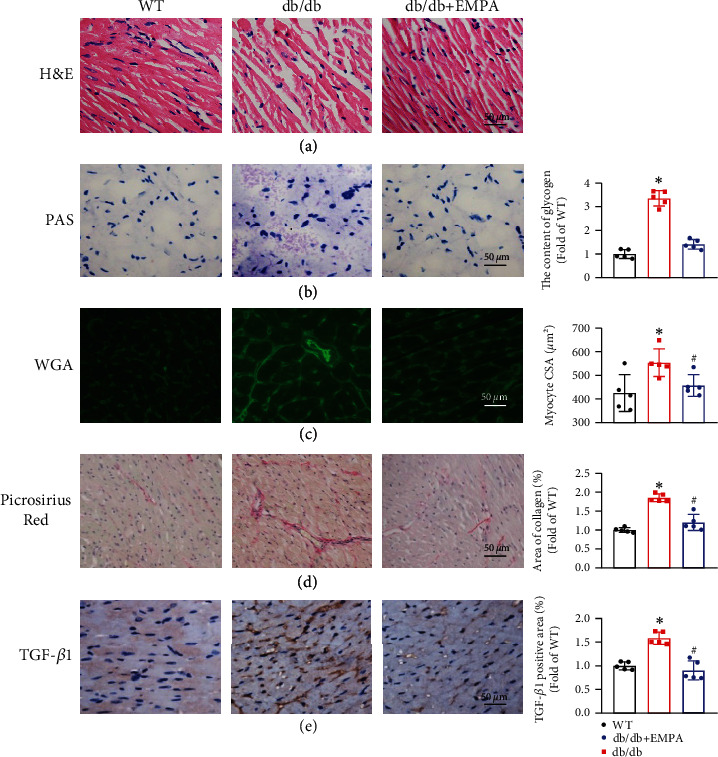
EMPA treatment prevented diabetes-induced myocardial pathological changes. (a) Representative images of H&E staining. (b) Glycogen accumulation in heart tissues was determined with PAS staining and the glycogen content was quantified. (c) Representative images and quantitative analysis of CSA of cardiomyocytes with WGA staining. (d) The fibrosis of heart tissues was evaluated by Picrosirius red staining of collagen accumulation (collagen is red), and the collagen content was calculated and compared. (e) The expression level of TGF-*β*1 was examined with immunohistochemical analysis of mouse heart paraffin tissue section and the percentage of TGF-*β*1-positive area was calculated and compared. Data are shown as mean ± SD, *n* = 5 per group, ^∗^*P* < 0.05 vs. WT mice group; ^#^*P* < 0.05 vs*. db/db* mice treated vehicle group.

H&E histological staining showed that whereas the myocardial fibers of WT mice are neatly arranged, the myocardial fibers of *db/db* mice are disordered. However, *db/db* mice treated with EMPA showed improvements in the arrangement of their myocardial tissue fibers compared to *db/db* mice in the vehicle group (Figure 3(a)).

We investigated the role of EMPA treatment on glycogen deposition in the heart tissue of *db/db* mice by PAS staining. As shown in Figure 3(b), *db/db* mice treated with vehicle had a greater level of heart tissue glycogen deposition (indicated in magenta) than WT mice, EMPA treatment effectively inhibited glycogen deposition.

FITC-conjugated WGA staining was used to determine myocyte CSA. As shown in Figure 3(c), myocyte CSA in *db/db* mice treated with vehicle was markedly larger than in WT mice. While EMPA treatment significantly prevents the increase of myocyte CSA of *db/db* mice, compared to *db/db* mice in the vehicle group (*P* < 0.05).

Sirius-red staining was conducted to evaluate collagen accumulation and the degree of fibrosis of cardiac tissues. This demonstrated that a large amount of collagen was deposited in heart tissues in *db/db* mice treated with vehicle, but the extent of collagen deposition was markedly lower in *db/db* mice treated with EMPA (*P* < 0.05, Figure 3(d)). In addition, the expression of the profibrotic mediator, TGF-β1, was higher in the heart tissue of *db/db* group mice treated with vehicle than that of WT mice. However, this increase was no longer present in *db/db* mice treated with EMPA (Figure 3(e)).

### 3.4. EMPA Ameliorates Oxidative Stress in *db/db* Mouse Heart Tissues and PA-Treated H9C2 Cells

**Figure 4:**
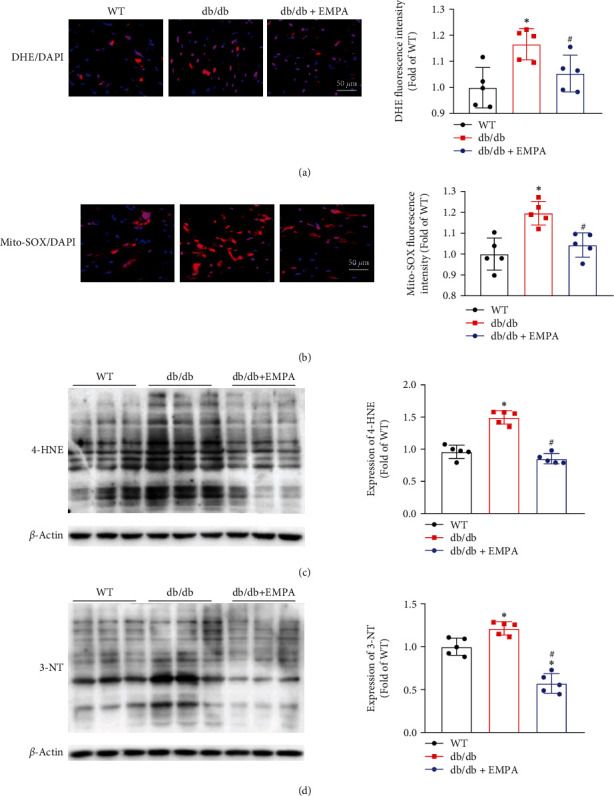
EMPA treatment reduces cardiac oxidative stress levels in *db/db* mice. (a) DHE staining was used to evaluate superoxide production in heart tissues. (b) Mitochondrial ROS levels in heart tissues were detected by Mito-SOX staining. The expression levels of 4-HNE (c) and 3-NT (d) in heart tissues were determined with western blot analysis. The data are expressed as mean ± SD, *n* = 5 per group, ^∗^*P* < 0.05 vs. WT mice group; ^#^*P* < 0.05 vs*. db/db* mice treated vehicle group.

Excessive oxidative stress in response to hyperglycemia and hyperlipemia is an inducer of DCM in mice [27]. The fluorescent probes DHE and Mito-SOX Red were used to measure the extent of accumulation of intracellular ROS or Mito-ROS in heart tissues, respectively. As shown in Figures 4(a) and 4(b), compared with WT mice, both intracellular and mitochondrial ROS levels were obviously increased in the heart tissues of *db/db* mice treated with vehicle. However, *db/db* mice treated with EMPA showed significantly reduced levels of ROS. Coinciding with these findings, the expressions of lipid peroxide 4-hydroxynonenalal and nitrosation production 3-nitrotyrosine, as well as oxidative stress-related markers, were considerably higher in *db/db* mice treated with vehicle compared with WT mice, but these changes were markedly attenuated by EMPA treatment (Figures 4(c) and 4(d)).

**Figure 5:**
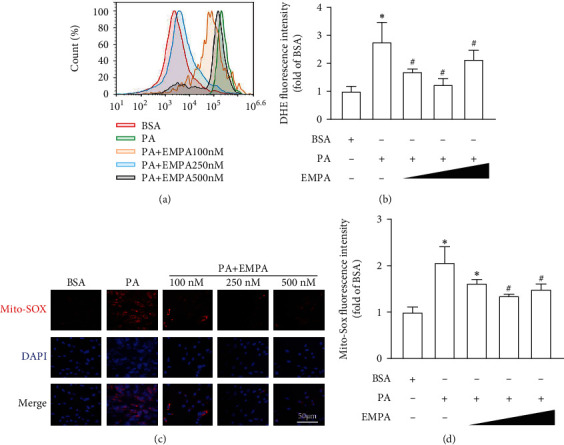
EMPA reduces PA-induced ROS production in H9C2 cells. H9C2 cells were exposed to 200 *μ*M PA with different concentrations (100 nM, 250 nM, and 500 nM) of EMPA for 24 h. (a) Intracellular ROS levels were assessed by DHE staining, and the fluorescence intensity of DHE was detected by flow cytometry. (b) Statistical values of average fluorescence intensity of DHE. Mitochondrial ROS levels were evaluated by Mito-SOX staining. (c) Representative image of Mito-SOX staining. (d) Quantification of fluorescence intensity of Mito-SOX. Three independent experiments were performed in each study. Data shown in graphs represent mean ± SD. ^∗^*P* < 0.05 vs. WT mice group; ^#^*P* < 0.05 vs*. db/db* mice treated vehicle group.

We further evaluated the effects of EMPA on oxidative stress by studying intracellular ROS and Mito-ROS levels in PA-induced H9C2 cells with or without EMPA treatment. The results showed that PA increases both intracellular and Mito-ROS levels, while EMPA treatment significantly attenuates these effects (Figure 5).

### 3.5. EMPA Improves Oxidative Stress by Activating the Nrf2 Signaling Axis

**Figure 6:**
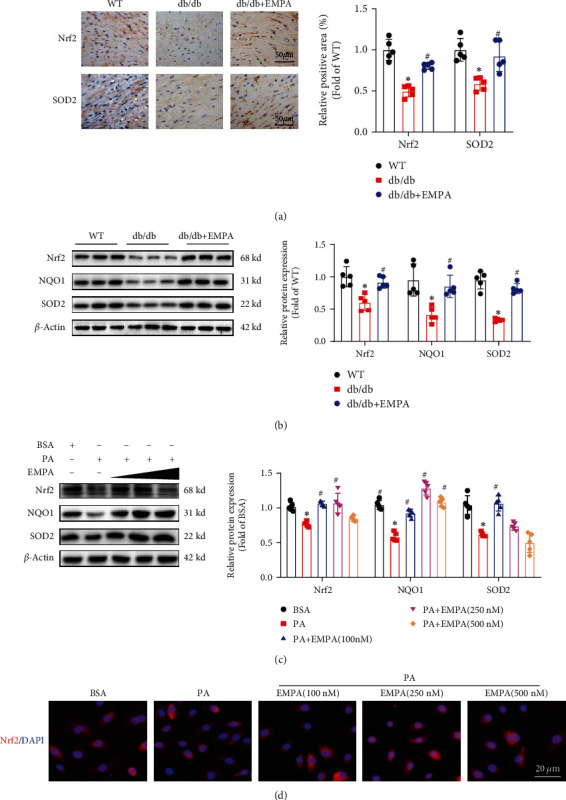
EMPA activates Nrf2 and upregulates its downstream antioxidant gene expression in the heart tissues of *db/db* mice and H9C2 cells treated with PA. (a) Immunohistochemical analysis of Nrf2 and SOD2 expression in heart tissues. (b and c) Nrf2 and its downstream target genes (NQO1 and SOD2) expression in heart tissues (b) and H9C2 cells (c) were detected by Western blot assay. (d) The expression and location of Nrf2 in H9C2 cells exposed to PA with different concentrations of EMPA were determined by immunofluorescence staining assay. Data are presented as mean ± SD, ^∗^*P* < 0.05 vs. WT mice group or BSA group; ^#^*P* < 0.05 vs*. db/db* mice treated vehicle group or PA group. For heart tissues analysis, *n* = 5 per group; For H9C2 cell analysis, three independent experiments were performed.

Nrf2 is crucial for oxidative stress regulation [28]. Thus, we investigated whether EMPA attenuates oxidative stress via activating the Nrf2 pathway. As shown in Figure 6(a), immunohistochemical analyses demonstrated the expression levels of Nrf2 and SOD2 were markedly decreased in heart tissues of *db/db* mice treated with vehicle compared with WT mice, EMAPA treatment prevented the decrease of Nrf2 and SOD2 expression. Western blot results also showed that the expression of Nrf2 and the target genes (NQO-1and SOD2) are decreased in the heart tissues of *db/db* mice treated with vehicle and H9C2 cells treated with PA. However, in both *db/db* mice and H9C2 cells treated with PA, EMPA treatment resulted in increased expression of Nrf2 and upregulated expression of NQO-1and SOD2 (Figures 6(b) and 6(c)). Furthermore, we evaluated whether EMPA promoted Nrf2 translocating from the cytosol into the nucleus via immunofluorescence staining assay. The results showed that EMPA treatment not only increase Nrf2 expression but also promoted Nrf2 translocating into the nucleus of H9C2 cells (Figure 6(d)).

### 3.6. EMPA Improves Mitochondrial Morphology and Function in the Heart of *db/db* Mice and PA-Treated H9C2 Cells

**Figure 7:**
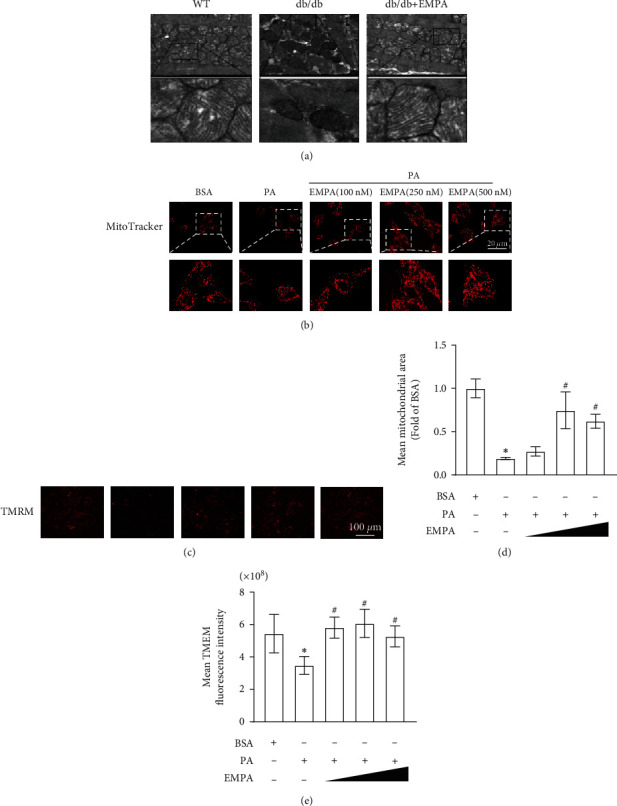
Effects of EMPA treatment on the mitochondrial morphology and membrane potential of H9C2 cells. (a) Representative TEM images of the heart tissues in WT or *db/db* mice. (b) Mitochondrial morphology was stained by MitoTracker® Red CMXRos probe and observed under a confocal laser microscope. (c) Representative images of the mitochondrial membrane potential determined by TMRE staining. (d) Calculations of mean mitochondria area. (e) Quantification of fluorescence intensity of TMRE in H9C2 cells under different treatment conditions. Data are shown as mean ± SD, ^∗^*P* < 0.05 vs. WT mice group; ^#^*P* < 0.05 vs*. db/db* mice treated vehicle group. For heart tissues analysis, *n* = 5 per group. For H9C2 cells, three independent experiments were conducted.

TEM was used to assess the effects of EMPA on mitochondrial morphology in heart tissues.  Figure 7(a) demonstrates the mean size of mitochondria of the heart tissues in *db/db* mice treated with vehicle is lower than that in WT mice, indicating that diabetes induces mitochondrial fragmentation. In contrast, EMPA treatment increases the size of mitochondria and inhibits mitochondrial fragmentation. Mitochondrial morphology in H9C2 cells was evaluated using MitoTracker® Red CMXRos probes. As shown in Figures 7(b) and 7(d), the mitochondria of H9C2 cells become smaller and shorter in length after exposure to PA, compared with the BSA group. However, when cells are treated with different concentrations of EMPA under PA condition, the mitochondria become filamentous and larger. In addition, EMPA treatment significantly inhibits the decline of the mitochondrial membrane potential levels induced by PA (Figures 7(c) and 7(e)).

### 3.7. EMPA Regulates the Mitochondrial Dynamics-Related Proteins in Myocardial Tissues and H9C2 Cells

**Figure 8:**
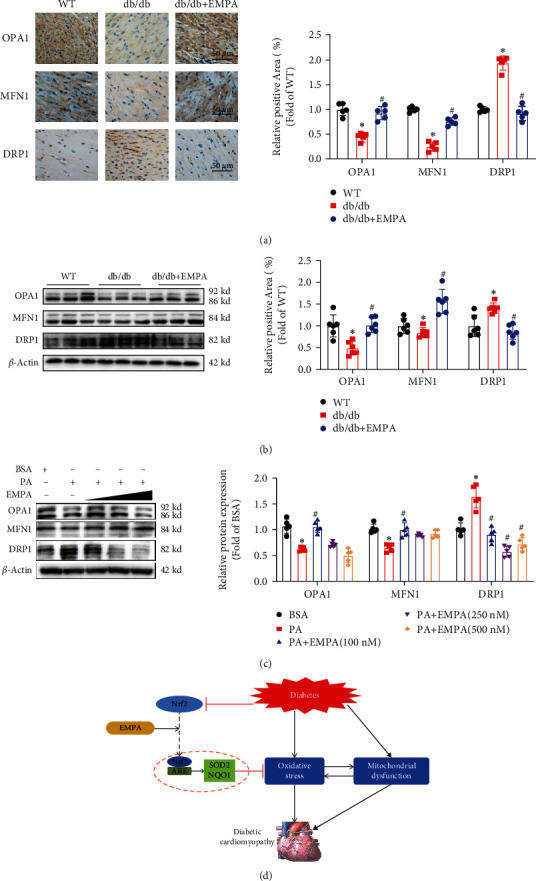
Effects of EMPA on the expression of proteins associated with mitochondrial fusion and fission in myocardial tissue and H9C2 cells. (a) Immunohistochemical staining was performed to evaluate the expression of OPA1, MFN1, and DRP1 in the heart tissues. (b and c) The expression of OPA1, MFN1, and DRP1 in myocardial tissue (b) and H9C2 cells (c) was evaluated with Western blot analysis. Data are shown as mean ± SD, ^∗^*P* < 0.05 vs. WT mice group or BSA group; ^#^*P* < 0.05 vs*. db/db* mice treated vehicle group, or PA group. For heart tissue analysis, *n* = 5 per group. For analyses in H9C2 cells, three independent experiments were performed. (d) Depicting diagram of the protective effects of EMPA on diabetic cardiomyopathy. Diabetes results in Nrf2 expression decrease in cardiomyocytes and induces oxidative stress and mitochondrial dysfunction. EMPA can rescue diabetes-induced inhibition of Nrf2 transcriptional activity, resulting in upregulation of antioxidant genes (SOD2 and NQO1), which attenuates oxidative stress induced by diabetes and improves mitochondrial function, ultimately rescues diabetic cardiomyopathy. ARE: antioxidant responsive element.

The above results suggested that EMPA is able to inhibit the mitochondrial fission induced by diabetes, meaning that EMPA influences mitochondrial dynamics. Mitochondrial dynamics are mainly mediated by the activity of the dynamin superfamily of large GTPases, DRP1, OPA1 and MFN1, and MFN2 [29]. Therefore, we examined the expression of factors involved in the regulation of mitochondrial dynamics in heart tissues and H9C2 cells. In heart tissues, the expressions of mitochondrial fusion-related proteins (MFN1 and OPA1) were greatly lower in *db/db* mice in the vehicle group than in WT mice, while EMPA treatment resulted in greater expression of OPA1 and MFN1 (Figures 8(a)–8(c)), but not MFN2 (data not shown). The expression of DRP1 was markedly higher in *db/db* mice in vehicle than in WT mice, but EMPA treatment prevented this increase (Figure 8(a)). Consistent with our results in heart tissues, PA treatment downregulated the expression of OPA1 and MFN1 and upregulated the expression DRP1 in H9C2 cells, but EMPA treatment upregulated the expressions of OPA1 and MFN1 and inhibited the increase of DRP1 expression induced by PA (Figure 8(b)).

## 4. Discussion

EMPA is a novel hypoglycemic agent in diabetic patients, showing the benefits for protection against the development of DCM. However, the exact functions and mechanisms by which EMPA protects against DCM are not been studied. Here, we evaluated the effects of EMPA on DCM in db/db mice and PA-treated H9C2 cells. Our results demonstrated that EMPA protected against diabetes-induced myocardial dysfunction, inhibited myocardial fibrosis and hypertrophy, and attenuated glycogen deposition in the hearts. Furthermore, EMPA treatment attenuated oxidative stress and improved mitochondrial function in heart tissues of db/db mice and H9C2 cells treated with PA. Finally, our results demonstrated that EMPA treatment reverses Nrf2 signaling in diabetic heart and H9C2 cells to attenuate oxidative stress and improve mitochondrial function through increasing the expression of Nrf2 and its downstream genes (NQO-1 and SOD2) (Figure 8(d)).

DCM is a common complication of diabetes. Myocardial fibrosis and hypertrophy are important pathological features of DCM, which reduce cardiac compliance, aggravate ventricular stiffness, and eventually lead to cardiac insufficiency [30]. The present study found that EMPA prevented DCM as reflected by the reduced myocardial collagen fiber area and glycogen deposition and inhibited myocardial hypertrophy. EMPA, as an SGLT-2 inhibitor, is a new class of drugs for the treatment of T2DM via increasing glucose excretion in the urine. In addition to its excellent glycemic effects, SGLT-2 inhibitors also have multiple protective effects on cardiac function via attenuating cardiac fibrosis, inflammation, and oxidative stress [31–33]. Nevertheless, SGLT-2 is predominantly expressed in the kidney, but not in other tissues such as the heart, which implies that the protective effects of EMPA on cardiac function are independent of organ-specific SGLT-2 expression [34–36]. Future studies need to be performed to elucidate whether the cardiac-protective effects of EMPA described here are dependent on the expression of the SGLT-2 receptor or not via experiments in SGLT-2 knockout or knockdown models.

Our findings reveal the potential mechanisms involved in EMPA's protective effects against DCM. Oxidative stress is caused by an imbalance between the production of oxygen/nitrogen free radicals and endogenous physiological antioxidant mechanisms. Oxidative damage caused by free radicals is considered an important factor that contributes to the development of T2DM and its complications. Growing evidence indicates excessive ROS levels in the heart of diabetes increase the risk of arrhythmia and mitochondrial dysfunction, which leads to myocardial cell necrosis and apoptosis [37–39]. In addition, ROS can also modify protein excitation-contraction coupling, activate hypertrophy signaling kinases, impair endothelial function and vascular homeostasis, and gradually exacerbate the damage associated with angiogenesis and new blood vessels information, eventually, resulting in cardiac dysfunction [1, 26]. Thus, alleviating oxidative stress is a potential strategy for preventing DCM. As expected, ROS and lipid peroxides were dramatically accumulated in the heart tissues of *db/db* mice and in PA-treated H9C2 cells, while EMPA treatment significantly attenuated the oxidative stress via inhibiting the accumulation of ROS and lipid peroxides. We also detected the dose-response of EMPA on H9C2 cells and found a medium dose (250 nM) of EMPA is more effective than a high or low dose, implying that the effects of EMPA are not dose-dependent. One reason for this may be that the high concentration of EMPA used here caused cytotoxicity. Thus, optimal doses of EMPA will need to be evaluated for clinical use.

Nrf2 is a basic leucine zipper stress-responsive transcription factor that plays a critical role in ameliorating oxidative stress by activating the expression of antioxidant proteins [40–43]. To investigate whether EMPA attenuates oxidative stress via activating the Nrf2 signaling pathway, we studied the expression of Nrf2 and its downstream antioxidant genes (NQO1 and SOD2) in the heart tissues and H9C2 cells. The results demonstrated that the decreased expression of Nrf2 and its downstream antioxidant genes in diabetes was reversed by the treatment with EMPA, indicating that the antioxidant effects of EMPA are largely through activating Nrf2 signaling. However, how EMPA activates Nrf2 requires further elucidation.

Mitochondria are the primary organelles of ROS production and are also the primary targets of the adverse effects of ROS. Exposure to high glucose conditions enhances mitochondrial ROS production and induces oxidative stress, which in turn impairs mitochondrial function [44]. Emerging evidence has confirmed that mitochondrial dysfunction is a critical contributor to the development of DCM [45, 46]. Mitochondrial function is reflected in mitochondrial structure and morphology. Mitochondria in T2DM patients are smaller than that in healthy controls; moreover, hyperglycemia can result in mitochondrial fragmentation in various cell types [13, 47, 48]. In our study, we found the size of mitochondria in the heart tissues of *db/db* mice is much smaller than that of WT mice. In addition, PA indues mitochondrial fragmentation in H9C2 cells, whereas EMPA treatment almost normalized the size of mitochondria in db/db mice and remarkably prevented this diabetes-induced mitochondrial fragmentation in cardiomyocytes in PA treated H9C2 cells. Mitochondrial morphology is dynamically regulated by mitochondrial fusion and fission, and several dynamin-related GTPases are involved in this process. In our experiments, EMPA significantly promoted the expressions of mitochondrial fusion-related proteins (MFN1 and OPA1) and also inhibited the expression of DRP1. These findings indicate that EMPA may ameliorate mitochondrial function by inhibiting the mitochondrial fission in the heart of diabetes. However, how EMPA regulates the expression of mitochondrial-related proteins is needed to be clarified in future.

## 5. Conclusion

In summary, this study indicated that DCM is associated with oxidative stress and mitochondrial dysfunction, and that EMPA protects against DCM through effectively inhibiting oxidative stress by activating Nrf2 and its downstream antioxidant genes and improving mitochondrial function via inhibiting mitochondrial fragmentation. Our present study provides a promising therapeutic approach for clinical translation to manage the progressive DCM in individuals with diabetes.

## Figures and Tables

**Table 1:** Biochemical assessments in experimental groups of mice.

	WT	*db/db*	*db/db*
Body weight (g)	21.33 ± 1.12	36.22 ± 1.86^∗^	35.29 ± 1.70^∗^
Body weight gain (g)	7.89 ± 1.54	22.11 ± 5.56^∗^	20.29 ± 3.04^∗^
Heart weight (mg)	161.13 ± 27.90	182.80 ± 25.24	168.10 ± 13.22
Heart weight/tibia length (mg/mm)	8.61 ± 1.56	10.69 ± 1.34^∗^	9.39 ± 1.51
Fasting blood glucose (mM,before treatment)	6.56 ± 2.53	15.94 ± 1.58^∗^	16.26 ± 2.79^∗^
Nonfasting blood glucose (mM,before treatment)	9.34 ± 0.79	24.46 ± 5.35^∗^	24.71 ± 1.91^∗^
Fasting blood glucose (mM,after treatment)	6.69 ± 1.56	21.10 ± 6.59^∗^	17.69 ± 6.30^∗^
Nonfasting blood glucose (mM, after treatment)	9.48 ± 0.68	28.42 ± 3.36^∗^	20.00 ± 2.49^∗^^**#**^
TC (mM)	4.08 ± 1.20	8.58 ± 1.23^∗^	5.27 ± 1.12^**#**^
HDL-C (mM)	2.59 ± 0.60	2.10 ± 1.26	3.94 ± 0.41^∗^^**#**^
LDL-C (mM)	1.00 ± 0.19	3.18 ± 0.56^∗^	218 ± 0.49^∗^
TG (mM)	1.24 ± 0.22	2.38 ± 0.72^∗^	2.11 ± 0.40^∗^

**Note:** Data are presented as mean ± SD. ^∗^*P* < 0.05 vs. WT mice group; ^#^*P* < 0.05 vs*. db/db* mice treated vehicle group. TC: total cholesterol; HDL-C: high-density lipoprotein cholesterol; LDL-C: low-density lipoprotein cholesterol; TG: triglyceride.

**Table 2:** Echocardiographic assessment of left ventricle structure and function in mice.

	WT	*db/db*	*db/db*+EMPA
HR (bpm)	538.84 ± 29.27	427.29 ± 60.03^∗^	470.74 ± 66.60^∗^
IVSd (mm)	0.41 ± 0.09	0.48 ± 0.08	0.51 ± 0.04^∗^
LVIDd (mm)	3.9 ± 0.31	4.14 ± 0.17^∗^	4.14 ± 0.39
LVPWd (mm)	0.47 ± 0.09	0.54 ± 0.06	0.51 ± 0.06
LVIDs (mm)	2.16 ± 0.29	2.55 ± 0.29^∗^	2.38 ± 0.36
EDV (ml)	0.16 ± 0.03	0.18 ± 0.02	0.18 ± 0.05
ESV (ml)	0.03 ± 0.01	0.05 ± 0.02^∗^	0.04 ± 0.02
LVEF (%)	81.71 ± 3.12	74.71 ± 7.08^∗^	79.52 ± 6.09^#^
LVFS (%)	44.60 ± 3.18	38.40 ± 5.54^∗^	42.79 ± 5.70^#^
SV (ml)	0.12 ± 0.02	0.13 ± 0.02	0.14 ± 0.04
CO (mL/min)	62.00 ± 13.98	56.25 ± 11.88	66.25 ± 15.06

Note: Data are presented as mean ± SD. ^∗^*P* < 0.05 vs. WT mice group; ^#^*P* < 0.05 vs*. db/db* mice treated vehicle group. HR: heart rate; IVSd: interventricular septal width during end-diastole; LVIDd: left ventricular internal diastolic diameter; LVPWd: left ventricular posterior wall diameter; LVIDs: left ventricular internal systolic diameter; LVEDV: left ventricular end-diastolic velocity; LVESV: left ventricular end-systolic volume; LVEF: left ventricular ejection fraction; LVFS: left ventricular fractional shortening; SV: stroke volume; CO: cardiac output.

## Data Availability

The data that support the findings of this study are available from the corresponding author upon reasonable request.
